# A novel LNG-IUS suture fixation method utilizing the hysteroscopy cold knife system: nail-type anchoring suture

**DOI:** 10.3389/fmed.2025.1714366

**Published:** 2026-01-12

**Authors:** Duan Duan, Li He, Chengling Zhang, Qiannan Hou, Qinyan Cao, Ying Xiong

**Affiliations:** Chengdu Women's and Children's Central Hospital, School of Medicine, University of Electronic Science and Technology of China, Chengdu, China

**Keywords:** fixation of LNG-IUS under cold knife system, improved intrauterine device anchoring, LNG-IUS fixation procedure, LNG-IUS nail-type anchoring suture, LNG-IUS suturing under cold knife system

## Abstract

**Background:**

The levonorgestrel intrauterine system (LNG-IUS) may have its contraceptive or therapeutic efficacy compromised if displacement or expulsion occurs. By suturing and securing the device to the uterine wall, the risk of expulsion can be significantly reduced. This approach ensures sustained contraceptive and therapeutic effects, particularly for patients with larger uterine volumes or a history of IUD displacement.

**Methods:**

Our team delineated the material selection criteria, operating system, and instrument configuration plan for LNG-IUS nail-type anchoring suture surgery via a clinical case, and illustrated the surgical operation steps in detail.

**Results:**

Through comparative analysis of patients' preoperative and postoperative conditions, integrated with intraoperative imaging and postoperative ultrasonographic data, this study confirms that the innovative nail-type suture technique for LNG-IUS has demonstrated the intended efficacy. It exhibits significant clinical utility, offering an efficacious therapeutic option for patients presenting with abnormally enlarged uterine cavities or recurrent LNG-IUS displacement or expulsion.

**Conclusions:**

Our team pioneered the innovative development of the nail-type anchoring suture technique. This approach departs from conventional suturing methodologies and existing modified fixation techniques. It achieves robust anchoring while obviating the necessity for hysteroscopic visualization during ring removal. This significantly improves clinical outcomes and yields substantial economic advantages.

## 1 Background

The levonorgestrel intrauterine system (LNG-IUS) is a device that uses the synthetic progestogen levonorgestrel as its active ingredient, achieving localized drug delivery through the sustained and slow release of the medication within the uterine cavity. Its core mechanisms of action include significantly inhibiting endometrial hyperplasia, thickening cervical mucus to impede sperm penetration, and partially suppressing ovulation. The primary indication for this system is long-term contraception, and it is also approved for treating gynecological conditions such as heavy menstrual bleeding, adenomyosis, and chronic pelvic pain (1). The 2023 SOGC Clinical Practice Guideline, designated as number 437, also advocates for the levonorgestrel-releasing intrauterine system (LNG-IUS) as a primary therapeutic intervention for the condition of adenomyosis (2). The LNG-IUD constitutes the primary therapeutic intervention for managing endometrial hyperplasia in the absence of atypia (3). The Levonorgestrel Intrauterine System (LNG-IUS) is emerging as an alternative therapeutic option for the treatment of endometrial hyperplasia and early-stage endometrial cancer in women who are not candidates for surgery (4). LNG-IUS has a wide range of therapeutic effects, thus garnering extensive attention in the field of gynecology.

LNG-IUS suture fixation through hysteroscopic suture is a revolutionary, minimally invasive approach (5). Compared to the current LNG-IUS suture fixation technique, the innovative nail-type anchoring suture technique designed by our team provides the advantages of convenient operation, minimal bleeding, and secure fixation. The principal benefit of the development and application of this surgical technique is that it obviates the necessity for direct visualization through hysteroscopy when patients require the subsequent removal of the levonorgestrel-releasing intrauterine system (LNG-IUS). This result aims to avoid uterine trauma caused by repeated intrauterine procedures or cervical dilation (6), while simultaneously reducing the costs associated with repeat hysteroscopic surgeries and anesthesia, demonstrating significant economic benefits.

## 2 Methods

### 2.1 Case data

**Figure 1:**
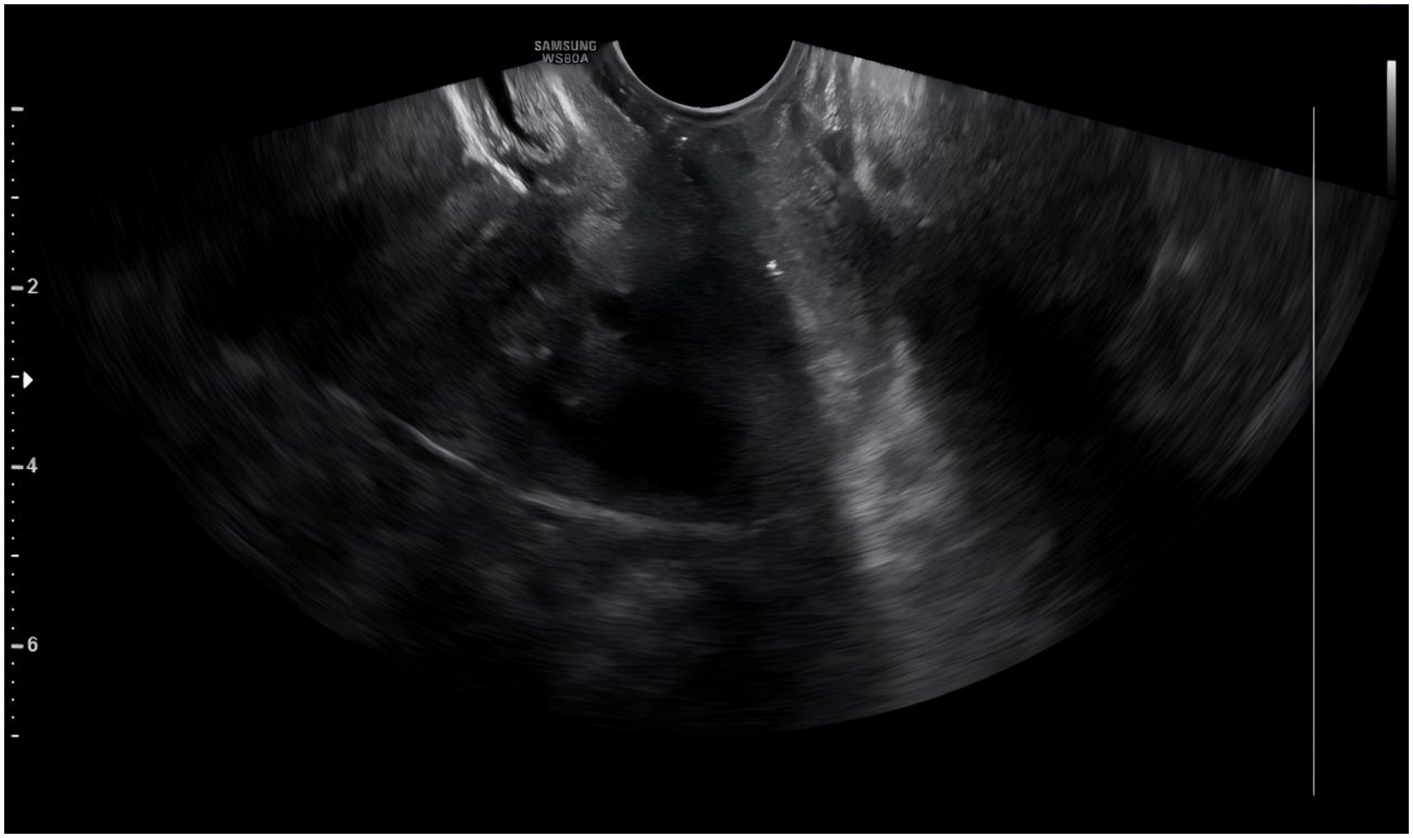
The patient's vaginal ultrasound indicates LNG - IUS displacement into the vagina.

The patient is a 38-year-old female who was admitted to our institution on April 23, 2025, presenting with “irregular vaginal bleeding persisting for over 9 months.” Nine months ago, she had an LNG-IUS inserted to address “abnormal uterine bleeding and adenomyosis.” Post-insertion, menstrual flow diminished; however, she experienced prolonged spotting for approximately 20 days, with menstrual intervals varying between 30 and 60 days. Her last menstrual period occurred on April 6, 2025. At the commencement of the current menstrual cycle, her flow was initially scant. Still, it intensified notably 2 days prior, accompanied by the passage of bright red blood clots, as well as symptoms of dizziness, fatigue, palpitations, and other discomforts. Upon physical examination at our facility, the IUD was visible at the cervical os without cervical motion tenderness, and the uterus was enlarged to the size of approximately 6 weeks' gestation. An outpatient ultrasound revealed adenomyosis, with the IUD not visualized intrauterinely but rather in the vaginal canal (Figure 1). The outpatient diagnosis was “abnormal uterine bleeding, adenomyosis, and LNG-IUS displacement,” which led to her hospitalization. The patient's surgical history encompasses two cesarean sections in 2007 and 2019, with bilateral tubal ligation performed concurrently with the 2019 procedure, one laparoscopic surgery for ectopic pregnancy in 2011, and a cholecystectomy for gallstones in 2022. No other significant medical history was reported.

Upon admission, the patient underwent a physical examination, which revealed stable vital signs, normal physical development, satisfactory nutritional status, and an active posture. Noteworthy was the presence of pallor, characterized by lips, eyelids, and nail beds that were slightly pale. The patient was alert, mentally sound, and exhibited cooperation throughout the examination.

The findings of the specialized examination were as follows: pubic hair was arranged in an inverted triangular pattern and was relatively dense. The external genitalia presented with characteristics of a married nulliparous individual, with traces of blood and no palpable masses. The vagina was unobstructed, devoid of mucosal congestion or any odor suggestive of Parazacco spilurus subsp. spilurus. The vaginal cavity contained fresh red blood and clots. A segment of the intrauterine device (IUD) tail was visible at the cervical os, with the IUD thread positioned at the vaginal lining. The cervix was smooth, of standard dimensions, non-tender upon palpation, and the cervical os was found to be closed. The uterus was enlarged, approximately corresponding to a 6-week gestational size, with a smooth surface and no tenderness. No masses or tenderness were detected in the bilateral adnexal regions.

Auxiliary examination: routine blood test indicates hemoglobin level of 90 g/L.

**Figure 2:**
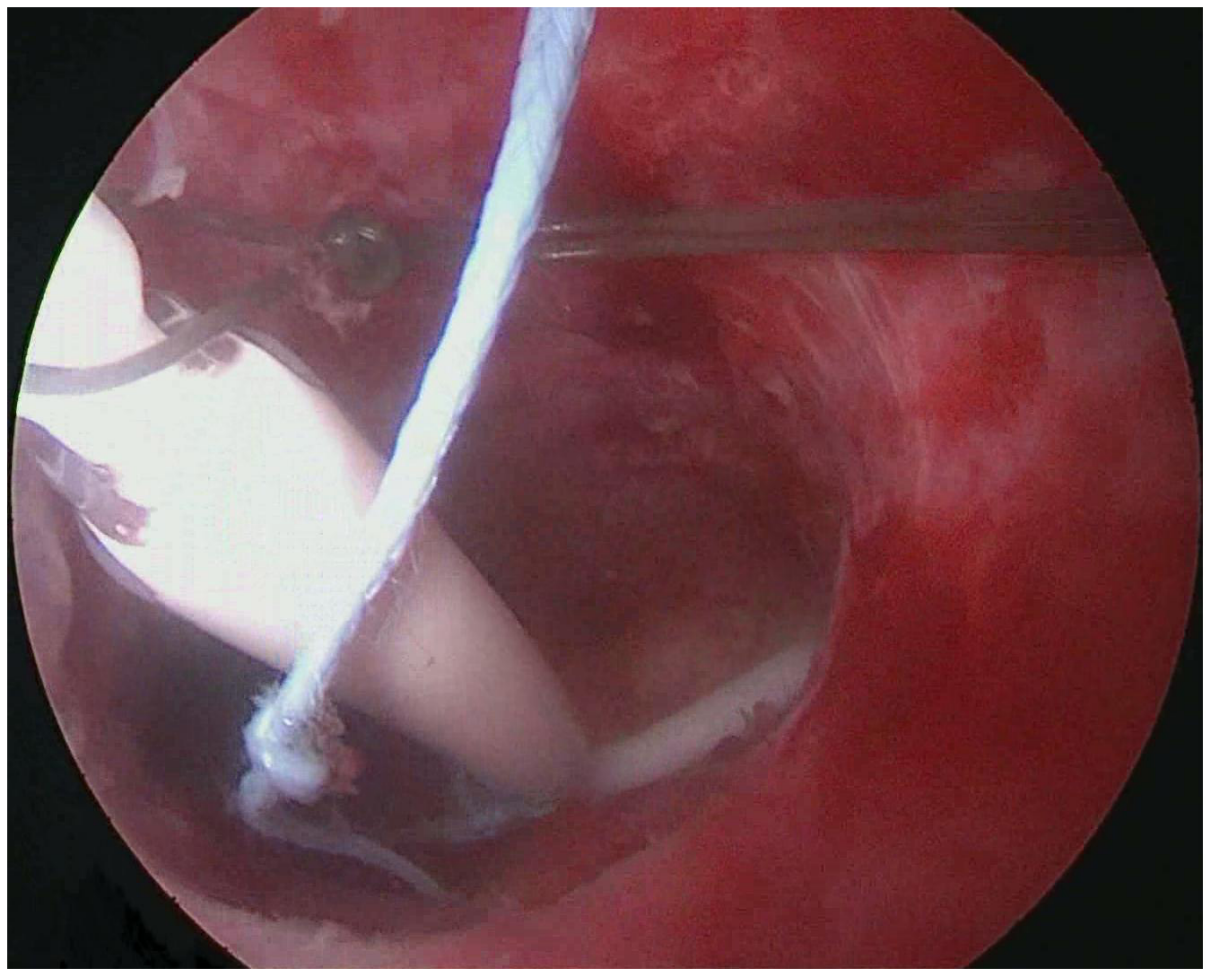
The LNG-IUS is placed in the uterine cavity, with both arms positioned at the uterine cornua, indicating normal placement.

**Figure 3:**
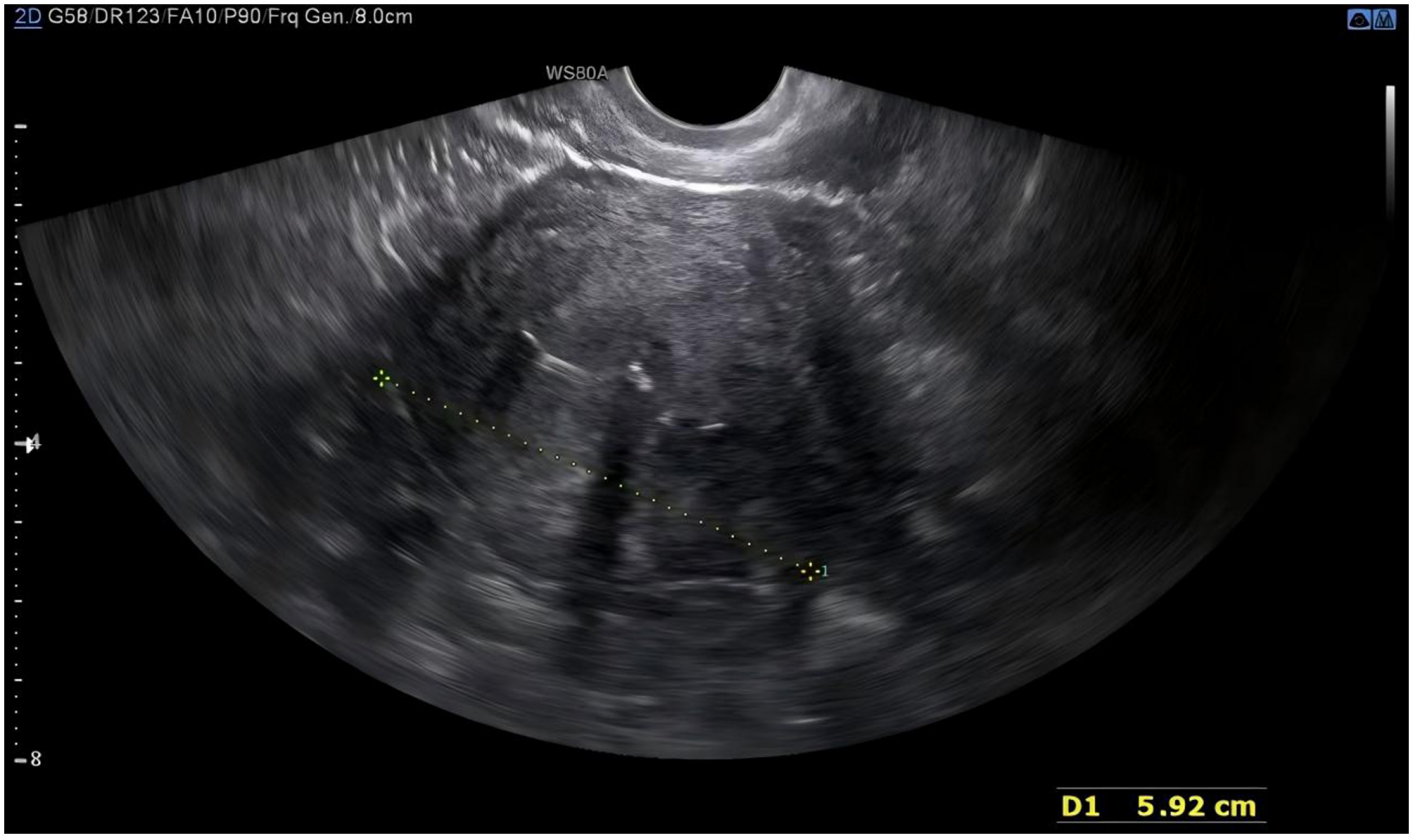
Post-operative vaginal ultrasound showed that the LNG-IUS was in a normal position within the uterine cavity.

Our team conducted a hysteroscopy on the patient. During the procedure, the uterus was found to be in an anteverted position, with the uterine cavity having a depth of 10 cm. Examination through the hysteroscope showed uneven endometrial thickness, and no intrauterine space-occupying lesion was detected. Subsequently, a diagnostic curettage was carried out. We then used a cervical dilator to dilate her cervical os to size 10 once again, followed by the insertion of a 25-fr cold knife system. The LNG-IUS was fixed using the LNG-IUS Nail Anchor Suture technique. After suturing, the LNG-IUS was positioned normally within the uterine cavity (Figure 2). Two weeks later, the patient underwent a follow-up ultrasound at the outpatient clinic, which confirmed the normal intrauterine position of the IUD (Figure 3).

### 2.2 Surgical methods and procedures

#### 2.2.1 Material selection

**Figure 4:**
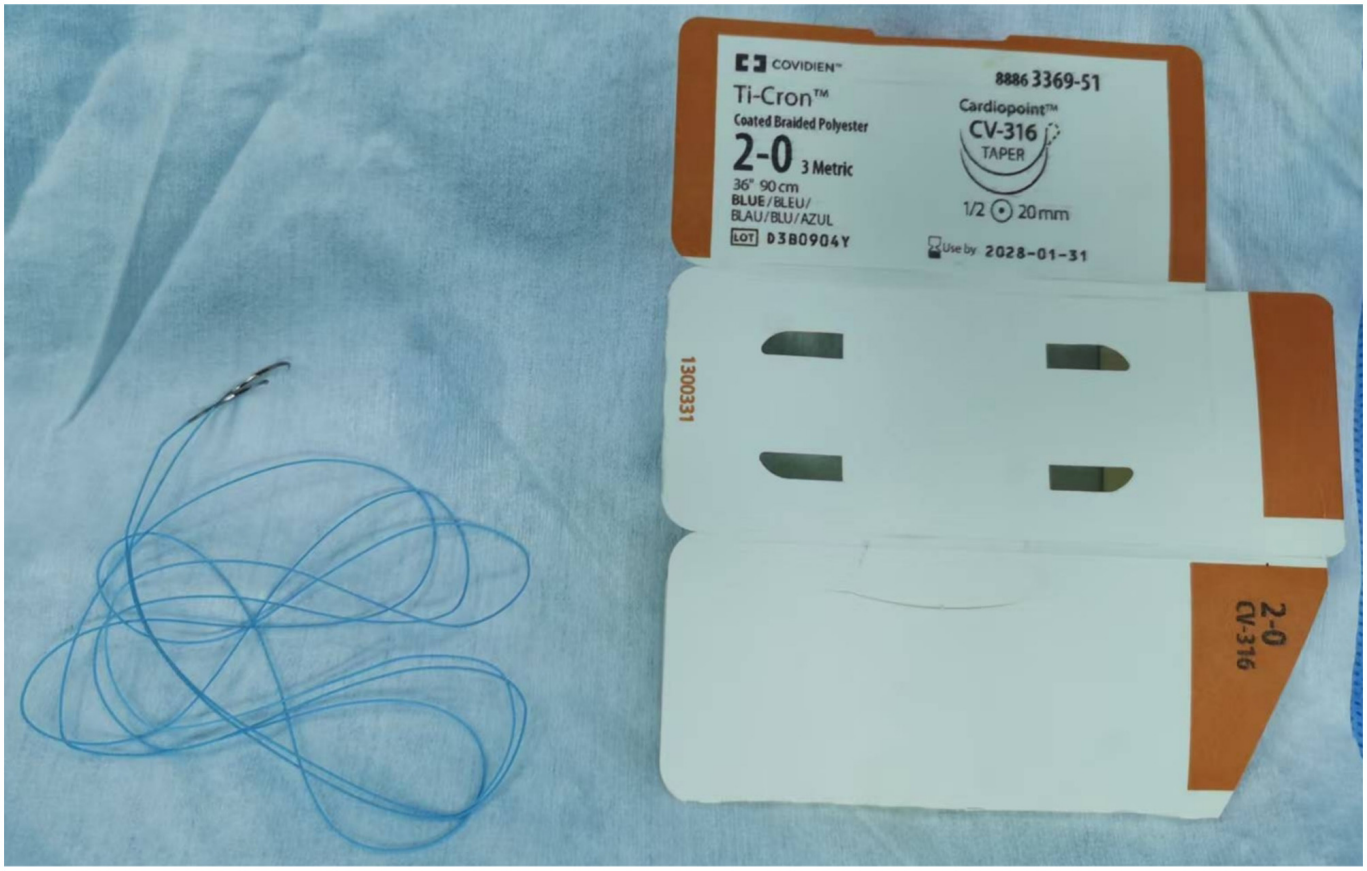
2-0 non-absorbable suture specification and 1/2 circle arc needle.

The selection of non-absorbable sutures is advised, with a preference for braided varieties. Braided sutures are constructed from interwoven multiple fibers, providing a textured surface that enhances friction with tissues, thereby ensuring a secure engagement during knotting. This results in a significantly diminished risk of postoperative knot slippage. Moreover, the pliable nature of braided sutures allows for a seamless adaptation to tissue contours, effectively reducing the potential for tissue laceration. The woven architecture, specifically that of Broussonetia papyrifera, ensures an even distribution of tensile forces across the individual fibers, bestowing upon them superior resistance to elongation. The chosen non-absorbable suture size is 2–0. The team has selected 1/2-circle needles, which possess a 180-degree curvature and are available in two lengths: 20 mm or 26 mm. It is preferable to have a higher needle body hardness, as this contributes to increased resistance against bending and deformation (Figure 4).

### 2.3 Selection of the cold knife system

**Figure 5:**
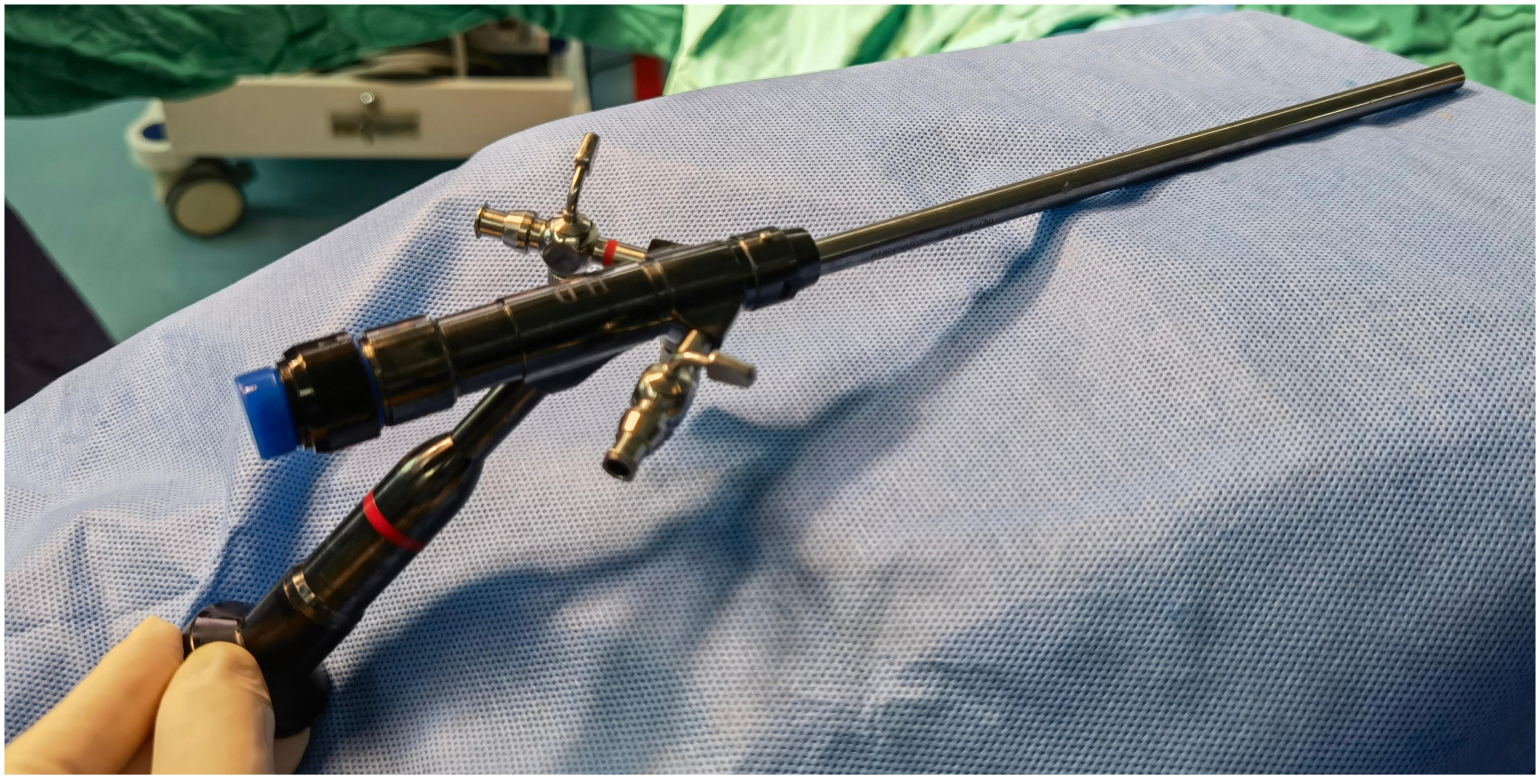
25fr cold knife system.

A total of 25-fr cold knife system: the working length of the operating sheath is 208 mm (Figure 5).

**Figure 6:**
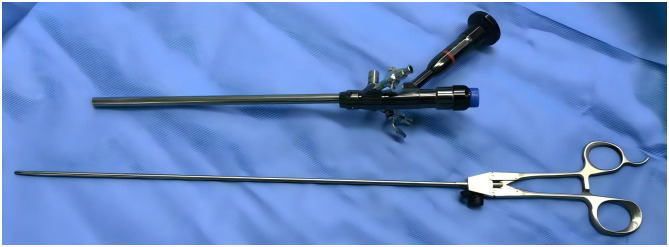
Needle holder compared with the 25fr cold knife system, the needle holder has sufficient operating length.

Needle holder: select a needle holder with a length of 36 cm and a diameter of 4 mm. This model offers a more stable and powerful grip when clamping 1/2—circle suture needles (Figure 6).

### 2.4 Specific steps of surgical techniques

#### 2.4.1 Anesthesia and positioning

General anesthesia was administered, either through tracheal intubation or via a combined spinal-epidural technique, with the patient subsequently positioned in the supine lithotomy posture. The cervical canal was dilated to facilitate the unhindered insertion of a size 10 cervical dilator into the uterine cavity. A continuous lavage with 9% saline solution was sustained, ensuring that the distension pressure remained at or below the mean arterial pressure.

#### 2.4.2 Operating procedure

**Figure 7:**
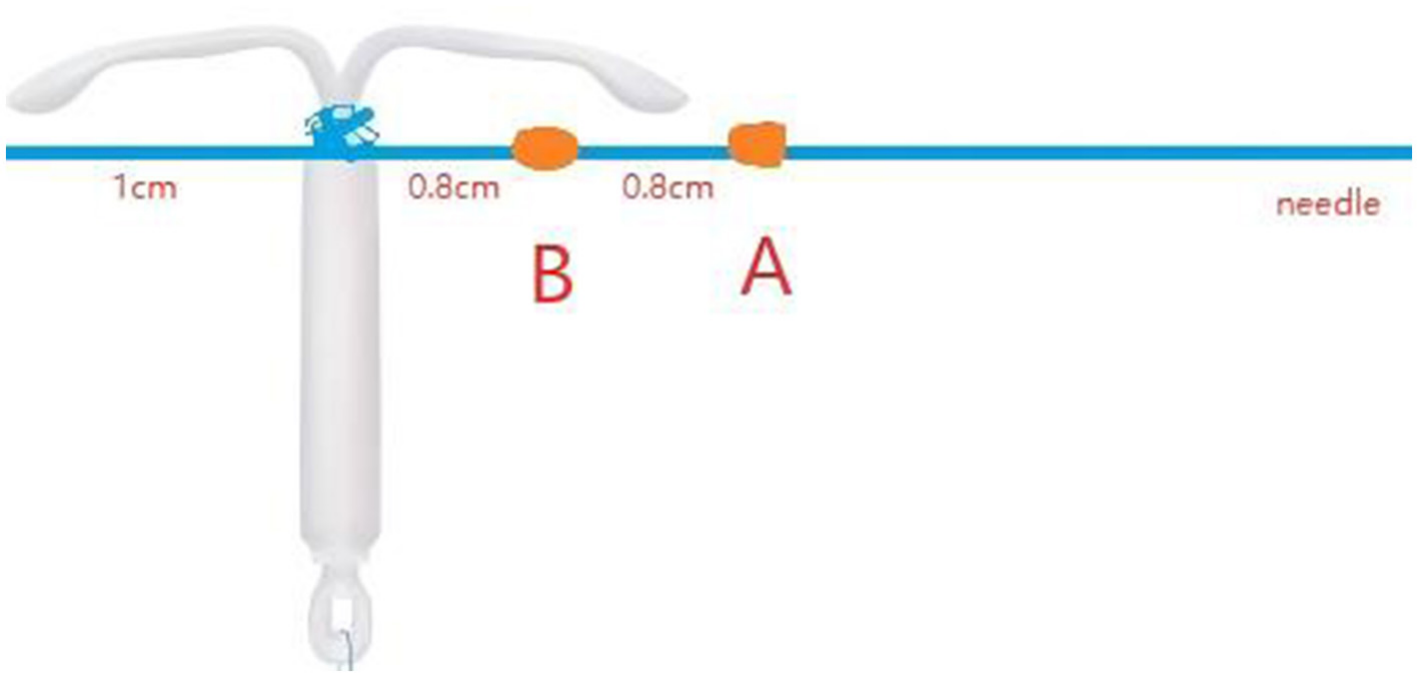
Schematic diagram of knotting positions between knot A and knot B.

**Figure 8:**
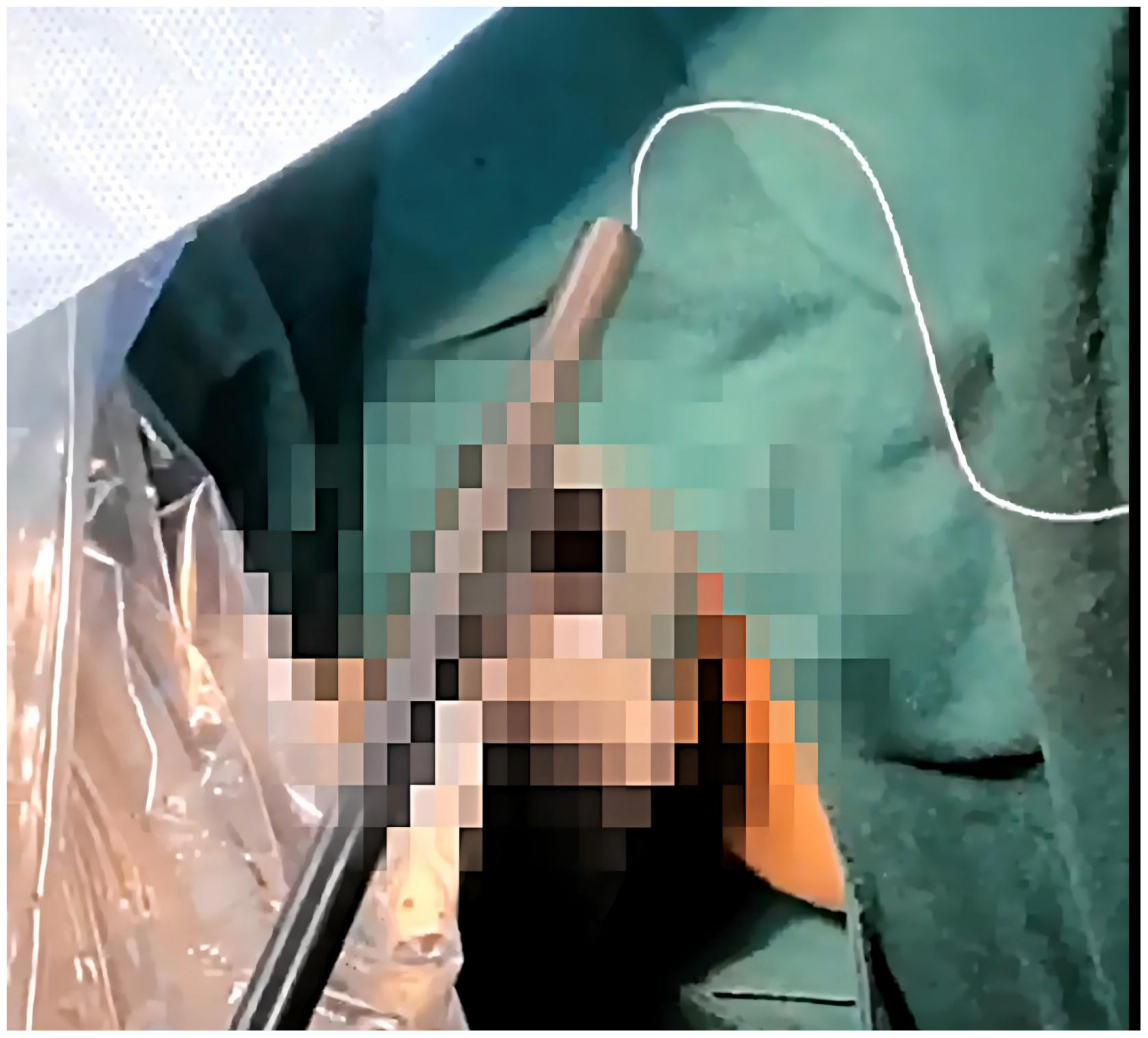
Before entering the uterine cavity, the needle should be withdrawn into the sheath.

**Figure 9:**
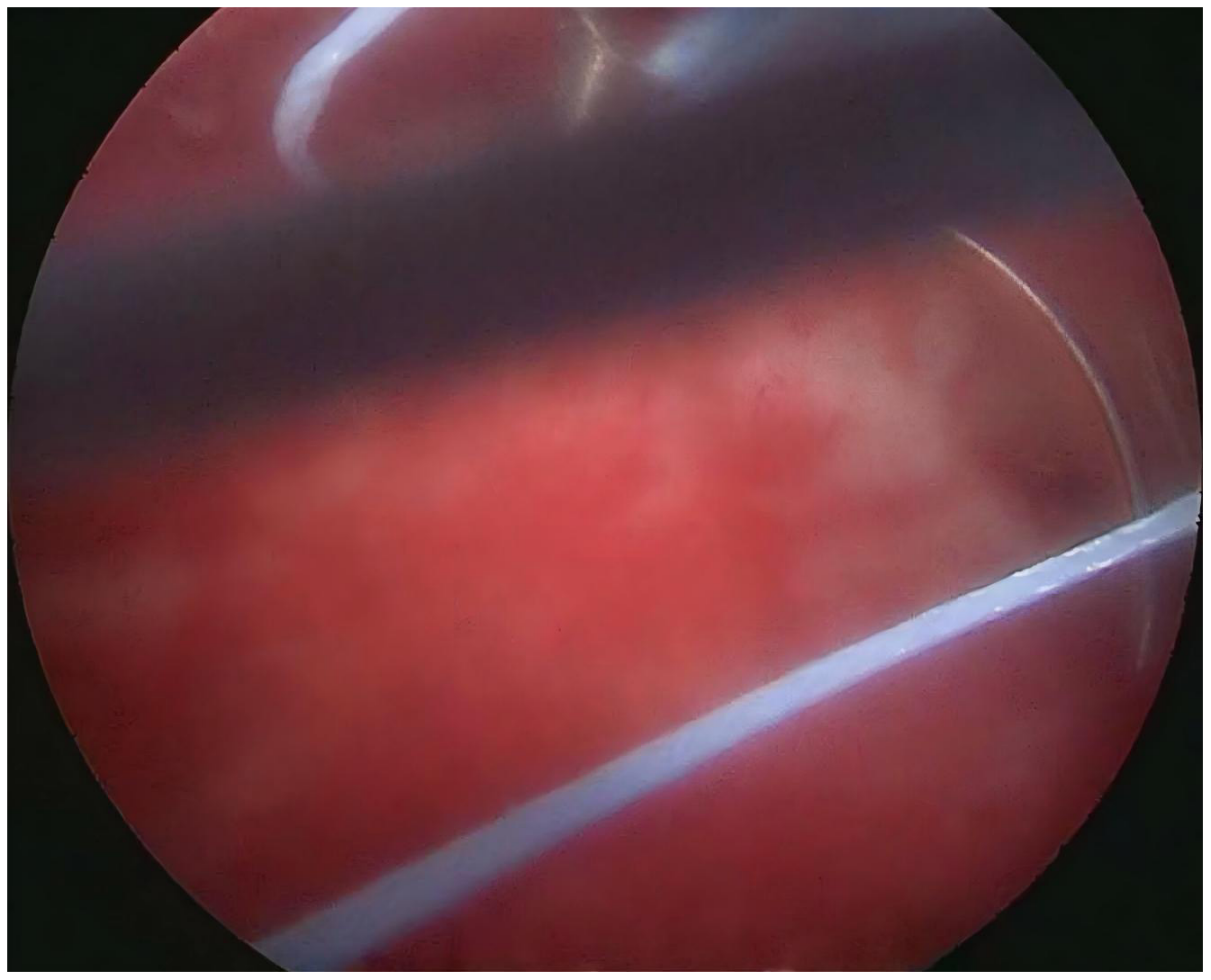
The needle tip vertically enters the posterior wall of the uterus.

**Figure 10:**
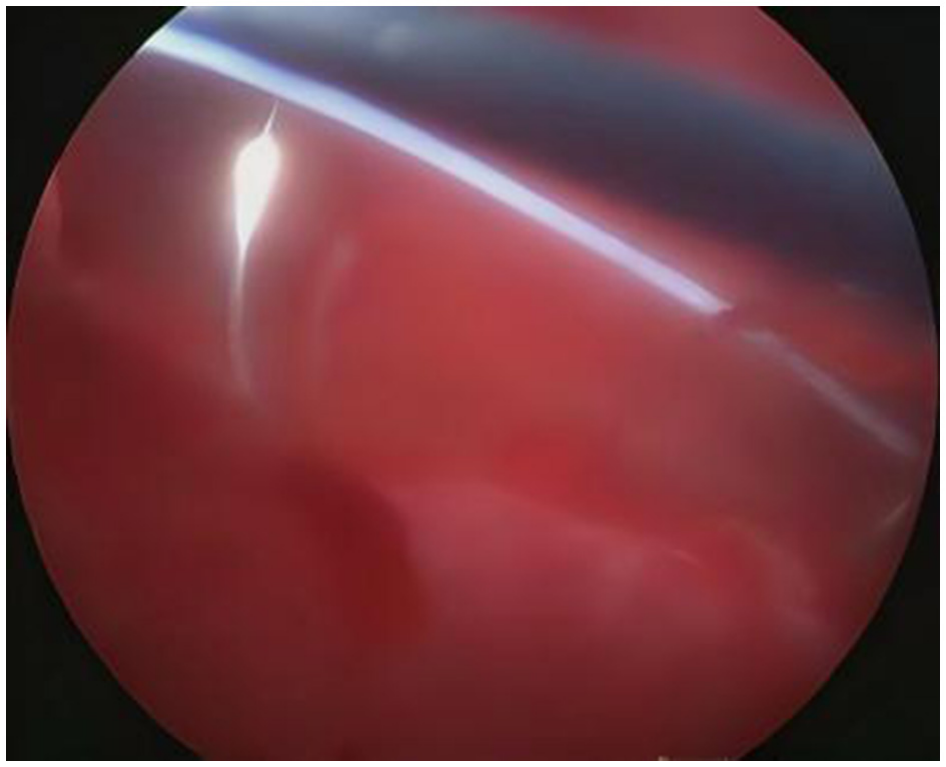
Withdraw the needle smoothly along its natural curvature.

**Figure 11:**
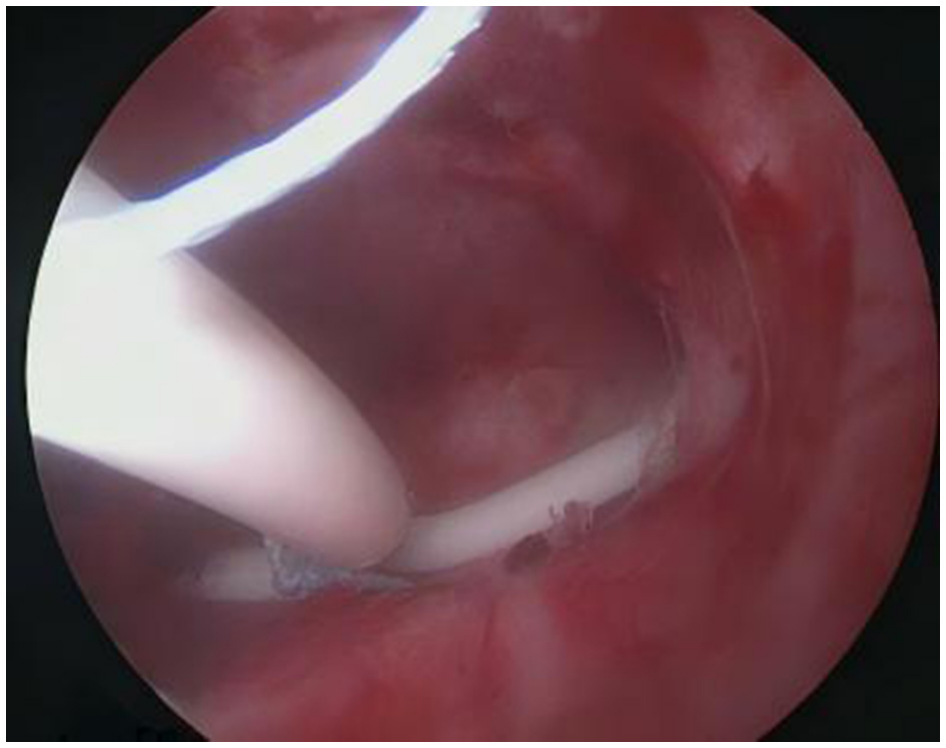
Knot A has been pulled out of the superficial muscle layer, so retain knot B as the anchor point within the superficial muscle layer.

**Figure 12:**
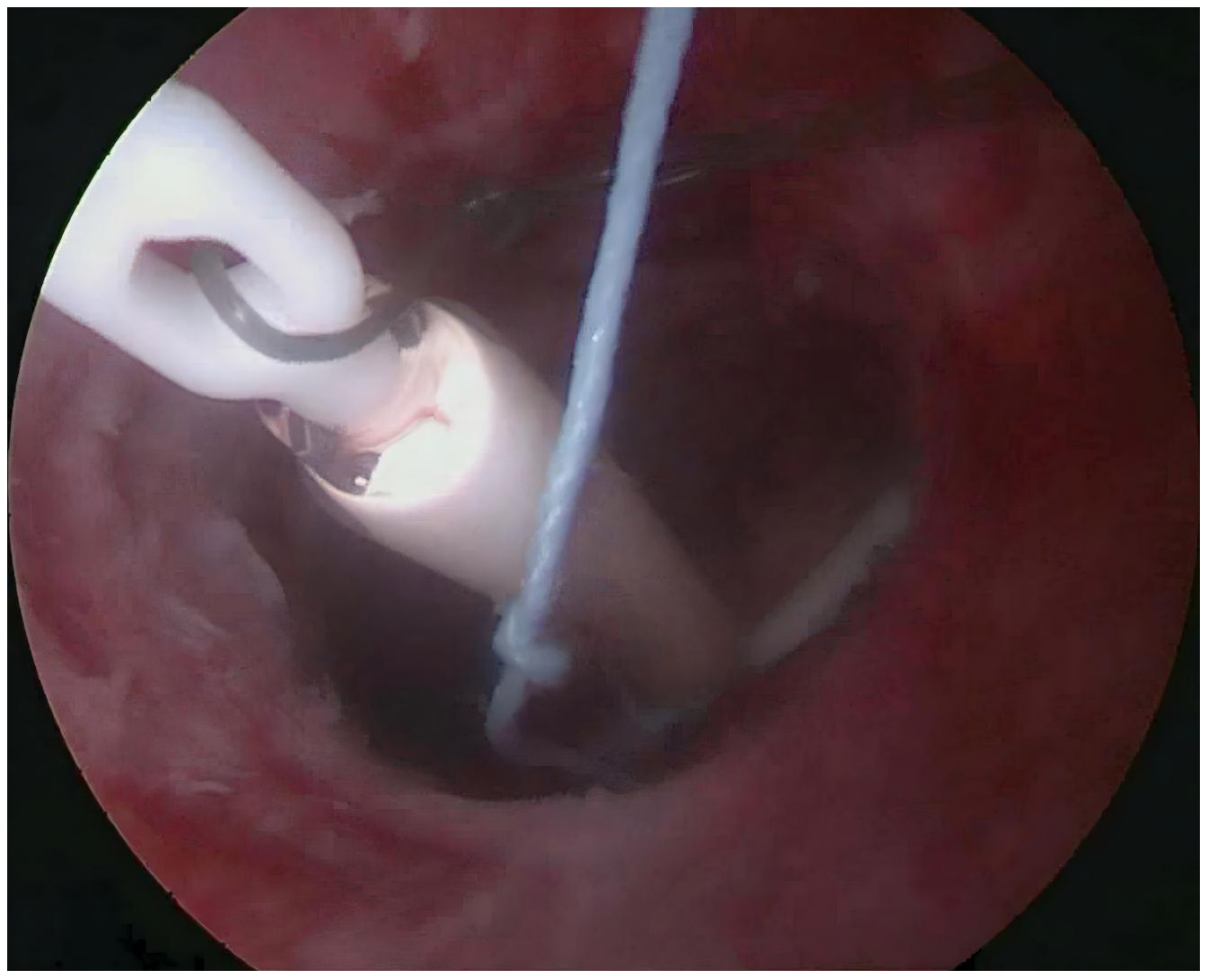
The LNG-IUS is correctly positioned within the uterine cavity, and the tail thread can be gently pulled out.

A. The steps for securing the reserved suture are as follows:
  First, create the first knot (referred to as Knot A) approximately 30 cm from the starting point of the suture needle. It is recommended to wrap the thread three times at this location to ensure the knot is secure and prevent it from loosening.
  Next, use the same method to create the second knot (referred to as Knot B) about 0.8 cm caudal to Knot A. Also, wrap the thread three times to enhance stability.
  Then, maintaining a 0.8 cm spacing caudal to Knot B (i.e., toward the tail end of the suture), fix the LNG-IUS at the junction between the ring arm and the body of the intrauterine device using a surgical knot technique with 6–7 wraps to ensure the contraceptive device is firmly secured.
  Finally, be sure to leave approximately 1 cm of suture tail at the end of the ring to prevent the contraceptive device from slipping off.
  Throughout the process, pay attention to maintaining the tightness of the knots and the accuracy of their placement (Figure 7).
B. We need first to perform hysteroscopy to rule out contraindications for LNG-IUS placement, then proceed with diagnostic curettage treatment, or perform operations such as uterine polyp removal.
C. Our team utilized the 25 fr cold knife system, grasped the suture with needle holders, retracted the needle back into the sheath, and placed the 2–0 non-absorbable suture into the uterine cavity under hysteroscopic visualization (Figure 8).
D. Using a 4 mm needle holder, vertically insert the needle into the posterior wall of the uterine fundus, then withdraw the needle following its natural curvature (Figures 9, 10).
E. Using needle holders to pull the thread from the needle side and extract it from the uterine cavity, adjust the arms of the LNG-IUS to be parallel with the uterine cornua using instrument forceps outside the body, then carefully insert it into the uterine cavity.
F. Under hysteroscopic visualization, we performed the string pulling operation. If both Knot A and Knot B were embedded in the superficial myometrium, the excess suture was trimmed. If Knot A had been pulled out of the superficial myometrium, Knot B was retained as an anchor in the superficial myometrium. At the same time, the excess suture was cut, thereby leaving Knot A within the uterine cavity (Figures 11, 12).
G. Under hysteroscopic visualization, we adjusted the position of the LNG-IUS to ensure its arms were fully expanded and closely adhered to both uterine cornua.
H. Ultimately, the tail thread is extracted, resulting in a 2 cm-long tail thread positioned at the external cervical os. Should the patient require subsequent removal of the ring, it can be accomplished by employing the standard method of pulling the tail thread.

## 3 Results

Our team has demonstrated the efficacy of the innovative nail-type suture technique for anchoring the levonorgestrel intrauterine system (LNG-IUS). Our team has successfully demonstrated the effectiveness of the innovative nail-type suture technique for securing the levonorgestrel intrauterine system (LNG-IUS) through a detailed case presentation and a comparative analysis of ultrasonographic imaging before and after treatment. This report meticulously outlines the criteria for selecting surgical materials, the principles governing the application of the cold knife system, and the adaptation strategy for specialized instruments. Additionally, it provides a comprehensive description of the operative procedure and critical technical aspects, illustrated with intraoperative photographs and schematic diagrams. The widespread adoption of this technique offers significant clinical and economic benefits, providing a dependable therapeutic solution for patients with uterine cavities that are too deep or who have experienced recurrent LNG-IUS displacement or expulsion.

## 4 Conclusions

Our research team has pioneered the groundbreaking development of the novel nail-type anchoring suture technique, which represents a significant advancement in gynecological surgical procedures. This innovative approach fundamentally differs from and improves upon traditional suturing methodologies as well as all previously existing modified fixation techniques. The technique's unique design enables it to achieve exceptionally strong and reliable tissue anchoring while completely eliminating the previously mandatory requirement for hysteroscopic visualization during intrauterine device removal procedures. This dual advantage not only dramatically enhances clinical outcomes by reducing procedural complexity and improving patient safety, but also generates considerable economic benefits by decreasing equipment costs and shortening operation times. The technique's superior performance characteristics and cost-effectiveness make it particularly valuable in resource-limited settings where advanced endoscopic equipment may not be readily available.

## 5 Discussion

The levonorgestrel-releasing intrauterine system (LNG-IUS) constitutes a highly efficacious form of contraception and gynecological treatment. Nonetheless, confident individuals, including those with uterine abnormalities, an abnormally large uterine cavity, or a history of expulsion of an intrauterine system, are at risk of experiencing displacement or expulsion of the device. Although the incidence of LNG-IUS displacement/expulsion remains comparatively low overall (at approximately 5%−10%), it is notably elevated in specific high-risk Homo sapiens populations. A clinical trial indicated that the risk of LNG-IUS expulsion increases with a uterine cavity depth greater than 9 cm and excessive menstrual bleeding (7). Intrauterine device (LNG-IUS) expulsion may trigger persistent symptoms and impose financial burdens on patients (9). Traditional placement methods struggle to effectively address such issues (10), thereby giving rise to the demand for fixation techniques. The fixed technique is designed to securely affix the LNG-IUS body (a T-shaped structure) to the endometrial or myometrial fundus via physical means, thereby preventing its dislocation or expulsion.

Building upon traditional hysteroscopic suturing methodologies, a multitude of refined suturing techniques have been developed (5, 8, 9), nevertheless, the drawback is that patients are required to undergo a hysteroscopic procedure for the removal of the LNG-IUS under direct visualization subsequent to a five-year period.

Later, anchoring techniques (1), and the tail wire of LNG-IUS were also introduced as fixation methods (10). The suture anchoring technique employing a nail-type device, as developed by our team, provides the benefits of secure suturing and resistance to detachment or displacement. Furthermore, it obviates the necessity for hysteroscopic removal of the device 5 years post-treatment, thereby circumventing the requirement for multiple intrauterine procedures and cervical dilation.

The indications for LNG-IUS nail-type anchoring suture are as follows:

a. Patients with a need for LNG-IUS treatment;
b. Uterine cavity depth greater than 9 cm;
c. Patients whose LNG-IUS is prone to expulsion or displacement due to excessive menstrual bleeding;
d. Patients with LNG-IUS expulsion or downward displacement occurring more than once;
e. Patients with recurrent endometrial polyps, combined with multiple uterine fibroids and an excessively large uterine cavity;

The contraindications for placement are the same as those for conventional LNG-IUS.

This surgical technique has initially shown its potential for feasibility and short-term effectiveness, especially among individuals with a history of recurrent displacement.

The levonorgestrel-releasing intrauterine system (LNG-IUS) is sutured to the shallow myometrium of the posterior uterine fundus, a pioneering LNG-IUS suturing technique. Compared with traditional methods, it has a shorter learning curve for surgeons, as evidenced by operative-time observations. Once more cases are collected, comparative studies will be done.

Economically, this method avoids hysteroscopic removal of the LNG-IUS after 5 years, saving hysteroscopy and anesthesia costs. The core of this paper is the proposal of a novel suturing technique; it's the first report on using a tack-anchoring method for LNG-IUS suturing, which solves expulsion problems and reduces medical costs. The “zero-to-one” breakthrough's value lies in showing feasibility rather than a large number of cases.

The patient in this report meets most LNG-IUS fixation indications. A 38-year-old with fertility requirements, she has adenomyosis, an enlarged uterine cavity, and a history of LNG-IUS displacement. After about 6 months of follow-up, the LNG-IUS stayed in place. She represents a large group of young women with adenomyosis and related conditions. This led to the innovative tack—anchoring suturing method, aiming to cut costs, lower expulsion rates, and help surgeons master the procedure quickly.

This paper presents a novel technical approach through case descriptions, images, and text, including surgical procedures, key technical points, and preliminary safety and efficacy data. It aims to provide a technical blueprint and encourage more centers to adopt this technique, validating its generalizability through multicenter studies.

In fact, the procedure has been completed in 11 patients, with one case involving expulsion of the intrauterine device (IUD). This patient had endometrial atypical hyperplasia and obesity [body mass index (BMI) of 41.7]. The depth of her vulvovaginal canal was 15 cm, while the depth of her uterine cavity was 8 cm, rendering surgical exposure and suturing extremely difficult. At the 1-month follow-up, the LNG-IUS ring was found to have been expelled. This case indicates that, in future prospective studies, patients with excessively high BMIs may need to be excluded to decrease the incidence of IUD expulsion or displacement.

During the surgical procedures carried out on these 11 patients, informed consent was obtained from all of them. Moreover, all patients consented to the public disclosure of their anonymized medical information and to the use of their anonymized data for scientific research and analysis.

We recognize the small sample size as a limitation and note it as a direction for future research. Based on this success and 10 previous cases, we plan a larger-scale prospective study.

Nonetheless, several challenges persist, including the lack of robust, evidence-based medical data, inadequate standardization of procedures, and limited long-term safety data. Subsequent research efforts should concentrate on the development of rigorous randomized controlled trials (RCTs), the creation of standardized operational procedures, the assessment of long-term outcomes, and the investigation of innovative fixation devices or designs. As technology progresses and evidence accumulates, it is anticipated that these methodologies will emerge as essential measures for ensuring the safe and efficacious application of LNG-IUS in high-risk Homo sapiens populations.

## Data Availability

The original contributions presented in the study are included in the article/supplementary material, further inquiries can be directed to the corresponding author.
